# Antibiotic Therapy for Chorioamnionitis to Reduce the Global Burden of Associated Disease

**DOI:** 10.3389/fphar.2017.00097

**Published:** 2017-03-14

**Authors:** Clark T. Johnson, Rebecca R. Adami, Azadeh Farzin

**Affiliations:** ^1^Division of Maternal-Fetal Medicine, Department of Gynecology and Obstetrics, Johns Hopkins University School of MedicineBaltimore, MD, USA; ^2^Department of Gynecology and Obstetrics, Johns Hopkins University School of MedicineBaltimore, MD, USA; ^3^Division of Neonatology, Department of Pediatrics, Johns Hopkins School of MedicineBaltimore, MD, USA; ^4^Department of International Health, International Center for Maternal and Newborn Health, Bloomberg School of Public Health, Johns Hopkins UniversityBaltimore, MD, USA

**Keywords:** chorioamnionitis, neonatal sepsis, intrapartum antibiotics, global maternal health, intraamniotic infection

## Abstract

Chorioamnionitis is associated with significant maternal and neonatal morbidity and mortality throughout the world. In developed countries, great progress has been made to minimize the impact of chorioamnionitis, through timely diagnosis and appropriate treatment. In the global setting, where many women deliver outside the healthcare facilities, this diagnosis is frequently overlooked and not properly treated. In addition to its impact on maternal health, a significant proportion of neonatal morbidity and mortality can be prevented by both recognition and access to readily available treatment. With the increasing focus on saving the most vulnerable members of society, we echo the need for providing parturient women with suspected chorioamnionitis universal access to appropriate therapy. We describe known effective antibiotic therapies for chorioamnionitis and provide an overview of additional potential antimicrobial treatments that might be effectively implemented in areas with limited access to care.

Chorioamnionitis significantly contributes to maternal and neonatal poor outcomes. It represents Intrauterine Infection and/or Inflammation (Triple-I) (Higgins et al., 2016). In the short-term, chorioamnionitis can be associated with maternal sepsis, multi-organ dysfunction, stillbirth and death. Among surviving newborns, chorioamnionitis is associated with asphyxia, and early onset neonatal sepsis (EONS) well as long-term neurologic sequelae including cerebral palsy (Johnson et al., 2014). Antibiotics have been the mainstay of intrapartum therapy, and proven to reduce associated maternal and newborn morbidity. Chorioamnionitis can be clinically diagnosed by the presence of maternal fever, prurulent discharge, and tachycardia in the mother or her fetus (Higgins et al., 2016). Additional diagnostic methods include placental or amniotic fluid evaluation, but have more limited clinical applicability. Timely diagnosis and appropriate treatment with empiric antibiotic therapy and fetal delivery improves maternal and neonatal outcomes. Unfortunately, throughout the world, access to antibiotics and treatment for chorioamnionitis is limited (Laxminarayan et al., 2016). A variety of antibiotics have been described for their use in similar infections. We present a summary overview of current standard of care antimicrobial therapy as well as additional antibiotics that can be considered to facilitate treatment in resource-limited settings.

## Maternal and neonatal morbidity associated with chorioamnionitis

Chorioamnionitis, or Triple-I, is associated with significant maternal morbidity including the need for cesarean delivery, uterine atony, postpartum hemorrhage, as well as post-partum wound infections (Johnson et al., 2014). Approximately, 1 in 30 deliveries is complicated by the presence of chorioamnionitis, however the prevalence increases with decreasing gestational age at the time of delivery and has been associated with an estimated 25% of preterm deliveries (Ericson and Laughon, 2015). In the absence of treatment, up to 1 in 5 newborns exposed to chorioamnionitis develop early onset neonatal sepsis (Chan et al., 2015). Definitive treatment is achieved with delivery of the uterine contents, while temporization with antibiotic therapy may permit trial of labor and vaginal delivery rather than immediate surgical cesarean delivery. Antibiotic therapy can improve fetal signs of infection such as fetal tachycardia and minimize some of the associated maternal morbidities (Johnson et al., 2014). Therefore, while antibiotic therapy cannot eradicate the infection, it is an important pillar of management to prevent exacerbation of disease prior to delivery (Higgins et al., 2016).

Fetal sequelae from chorioamnionitis span from acute short-term disease to life-long morbidities (Johnson et al., 2014). The Fetal Immune Response Syndrome (FIRS) contributes to the significant morbidity of the condition, suggesting that even with antibiotic treatment of the offending organism, in some cases the inflammatory response may still cause significant morbidity, and is a major cause of stillbirth or neonatal death (Gibbs, 2002; Kallapur et al., 2014). Early onset neonatal sepsis can result in multi-organ dysfunction including life-threatening symptoms of respiratory distress and hemodynamic instability, and can be associated with chronic lung disease and neurologic injury among survivors. These complications are exacerbated among preterm infants and likely account for a significant proportion of global neonatal mortality, with urgent need for research to better delineate its impact are urgently needed (Lozano et al., 2012). It is important to note that routine administration of antibiotics in some cases of spontaneous preterm birth (e.g., preterm premature rupture of membranes) has the potential to mitigate neonatal sequelae (Cousens et al., 2010). Chorioamniontis in the global setting is a potential cause of stillbirth (Gibbs, 2002). Therefore, proper diagnosis of chorioamnionitis, and prompt administration of antibiotics to the laboring mother can temporize management until delivery, or in some cases during referral and triage to a tertiary care center and has the potential to avert death and long-term morbidity in a significant number of women and children (Gülmezoglu et al., 2016).

The burden of chorioamnionitis is most profound in Low and Middle Income Countries (LMIC) (Chan et al., 2016). Worldwide, an estimated 40 million births occur at home, mostly in LMIC and usually in the absence of skilled birth attendants and without access to proper preventative care as well as diagnostics and proper treatment for possible infection (Liu et al., 2016). A combination of poor conditions and poor hygiene contribute to neonatal mortality, with evidence that clean delivery practice has the potential to almost halve the risk of neonatal mortality (Seward et al., 2012). Neonatal infections, prematurity, and intrapartum related events account for the vast majority of the neonatal deaths worldwide. In the presence of overt chorioamnionitis, timely administration of antibiotics has the potential to minimize the risk of overt maternal or fetal sepsis (Chan et al., 2015) and has been associated with a 40% reduction in the neonatal infectious sequelae (Tita and Andrews, 2010).

## Existing guidelines for treatment of suspected chorioamnionitis

General guidance for treatment for chorioamnionitis includes antibiotic coverage of the causative pathogens (Higgins et al., 2016). While widely subject to demographic and geographic variability, common organisms associated with chorioamnionitis include Ureaplasma (47%), Mycoplasma (30%), Gardnerella vaginalis (25%), bacteriodes (30%), gram negative rods including Escherichia Coli (8%), and Group B Streptococcus (15%) (Tita and Andrews, 2010). GBS specifically has wide variability in prevalence among global populations (Le Doare and Heath, 2013). The common feature of these organisms is that they represent genital microbes that can ascend and cause a strong inflammatory response.

Based on our knowledge of causative pathogens, a variety of antibiotic regimens are used to cover the presumed bacterial etiology of chorioamnionitis (Greenberg et al., 2012). However, current consideration for antimicrobial treatment for chorioamnionitis includes a combination of an aminopenicillin and gentamicin, with clindamycin or metronidazole generally added when cesarean delivery is performed (Tita and Andrews, 2010; Higgins et al., 2016). Of interest, neither antibiotic provides coverage against mycoplasma, which is a common organism associated with chorioamnionitis (Tita and Andrews, 2010). There are to date limited randomized controlled trials to evaluate superiority of antibiotic regimens to treat amniotic infections during ongoing labor to demonstrate effectiveness (Chapman et al., 2014). This suggests an absence of evidence basis for recommended type of treatment for clinical chorioamnionitis in a limited resourced setting. Therefore, current section of antibiotic regimen is driven not by absolute science, but limitations with existing research to demonstrate treatment superiority or non-inferiority.

Treatment of chorioamnionitis is challenged by accurate diagnosis, using clinical judgment in the assessment of signs of maternal fever, maternal and/or fetal tachycardia, and purulent cervical discharge (Johnson et al., 2014). Isolated maternal fever may arise from other causes, and is not necessarily an indication for diagnosis and treatment of chorioamnionitis (Higgins et al., 2016). Diagnosis can also be made on histologic placental evaluation after delivery, as well as microbial testing of amniotic fluid, each with significant limitations regarding clinical utility, particularly in a low resource setting (Johnson et al., 2014). Additional, there appears to be imperfect correlation between these diagnostic methods (Chan et al., 2016). Although chorioamnionitis is clearly associated with preterm labor and delivery, the evidence does not support the routine administration of antibiotics to women in preterm labor with intact membranes in the absence of overt signs of infection, with caution that such prophylaxis may exacerbate poor outcomes (Subramaniam et al., 2012). Antibiotics for preterm premature rupture of membranes are indicated and effective in reducing the risk of a number of early morbidities, including respiratory distress syndrome and infection, without having a significant impact on mortality (Cousens et al., 2010). The burden of neonatal disease due to Group B streptococcal infection has seen dramatic improvement with standardized treatment following universal screening in pregnancy (Koenig and Keenan, 2009). It remains universally sensitive to penicillin, with alternative antibiotic regimens considered only in cases of allergy. Therefore, intrapartum antibiotics to prevent EONS are effective and have reduced substantially the incidence of EONS in countries where they are implemented (Dutta et al., 2010).

## Additional antibiotics that can be considered for treatment of suspected chorioamnionitis

A number of antibiotics might be considered for use in pregnancy to treat amniotic infection, appreciating those listed in the WHO Guide of Essential Medications (Table 1) (WHO, 2016). Considering fetal organ immaturity and/or ongoing organ development, initial evaluation must consider potential fetal organ toxicity or teratogenic effects, even when given just prior to delivery. It is important to note, that while pregnancy category may not strictly contradict administration, other considerations might preclude their use as with fluoroquinolones (ACOG, 2016). Secondary considerations would include route of administration. This would be particularly relevant for developing regimens that can be implemented in LMIC. Therefore, many of these agents would be reasonable potential candidates in LMIC. As challenges remain in recommendation of superior treatment regimens for chorioamnionitis when compared to others, consideration of a variety of agents might be considered to provide effective treatment for chorioamnionitis in a low resource setting. Consideration of Trimethoprim/Sulfa for use is considered in light of potential association with kernicterus in neonates (Thyagarajan and Deshpande, 2014).

**Table 1:** **A selected list of antibiotics and routes of administration, as included in the WHO guide of essential medications (WHO, 2016), with pregnancy category, half-life, and indication of placental passage efficacy**.

**Antibiotic class**	**Antibiotic**	**Pregnancy category**	**Route**	**Half-life (hours)**	**Placental transfer**
Penicillins	Benzyl PCN	B	Injection	0.5	Incomplete
	Benzathine PCN G	B	Injection	30–50	Incomplete
	PCN V	B	Oral	0.5	Incomplete
	Procaine PCN G	B	Injection	20–40	Incomplete
Aminopenicillins	Ampicillin	B	Injection	1	Complete
	Amoxicillin	B	Oral	1.3	Complete
Penicillins: (Pellicinase Resistant)	Cloxacillin	B	Oral, Injection	0.5	Incomplete
Cephalosporins	Cefazolin	B	Injection	2	Complete
	Cephalexin	B	Oral	1	Complete
Cephalosporins:	Ceftriaxone	B	Injection	8	Complete
3rd Generation	Cefotaxime	B	Injection	1	Complete
	Ceftazidime	B	Injection	2	Complete
Vancomycin	Vancomycin^*^	C	Injection	4–6	Incomplete
B-Lacatmase Inhibitors	Amoxicillin/Clavulanate	B	Oral	1.0	Complete
Carbapenams	Imipenam+Cilastin	B	Injection	1–2	Incomplete
Aminoglycosides	Gentamicin	C	Injection	2–4	Incomplete
Macrolides	Erythromycin	B	Oral, Injection	1–1.5	incomplete
	Azithromycin	B	Oral	12	Incomplete
	Clarithromycin	C	Oral	5–7	Complete
Chloramphenicol	Chloramphenicol	C	Oral, Injection	1.2	Complete
Lincosamide	Clindamycin^*^	B	Oral, Injection	2–3	Complete
Fluoroquinolones	Ciprofloxacin	C	Oral, IV	3.7	Incomplete
Nitroimidazole	Metronidazole		Oral, injection, suppository	9	Complete
Nitroheterocylic	Nitrofurantoin	B	Oral	0.33	Incomplete
	Spectinomycin	B	Injection	2	Incomplete
Anti-Folate Agents	Trimethoprim/Sulfa	C	Oral, Injection	12	Incomplete
	Trimethoprim	C	Oral	12	Incomplete
Tetracycline	Doxycycline	D	Oral	12–16	Complete

*Adapted in part from Grayson et al. (2010), Roberts et al. (2008), and WHO (2016)*.

*PCN, Penicillin*.

* *Indicates WHO complementary medication, to be considered for specific clinical circumstances*.

The WHO list of essential medications provides guidance on a number of antibiotics that are most safe, efficacious, and cost-effective for priority conditions (WHO, 2016). Potential candidates for antibiotic therapy included on this list are further described with their half-life as well as relevant pharmacokinetic data on placental cross over (Table 1). Although treatment for chorioamnionitis before delivery tends to leave a short interval prior to delivery, duration of medication in case of needed repeat dosing may be considered. Given that chorioamnionitis represents an intra-amniotic infection, consideration of placental transmission of the medication would seem relevant. For each medication, individual considerations should be taken into account among other factors. Such factors include the compound stability, ease of administration, and medication cost, all of which are considered in the creation of the WHO list of essential medications. These factors may largely depend on geographic or local factors specific to communities. Additionally, medications that require weight-based considerations for dosing, such as aminoglycosides, macrolides, or vancomycin, might be challenging to implement. B-lactam antibiotics warrant consideration for increased dosing for particularly obese patients, which might be considered where obesity is a particular issue (Pevzner et al., 2011). Cultural acceptability of a regimen is important prior to implementation.

Accepted therapeutic regimens including ampicillin and gentamicin have similar antibiotic coverage compared to extended B-Lactamase agents (e.g., ampicillin/sulbactam). While multiple agents permit extended coverage, single agent regimens would seem to at least provide a modicum of convenience if not pragmatic integration in lower resource areas. Oral agents would have to consider their onset to action and bio-availability relative to IM or IV dosing, as time-conscious treatment of chorioamnionitis remains important. IV dosing will have pragmatic limitations in most communities. Cost will also have significant implications for universal provision, which would contribute to considering one agent over another.

## Steps to implementation of universal treatment for chorioamnionitis

Globally, there remains an unmet need of intrapartum treatment of chorioamnionitis. Many steps will be made before this need can be met. Antibiotic therapy will need to be affordable, transportable, and readily administered. In addition, regimens will need to demonstrate non-inferiority to standardized regimens. Universal birth attendance is an unmet goal in many LMIC. As efforts bend to millennium development goals help improve the safety of childbirth, attention will move to significant causes of morbidity such as chorioamnionitis. Just as with uterotonics for postpartum hemorrhage and magnesium for seizure prophylaxis, antibiotic therapy for intrapartum infection will be necessary to reduce the morbidity of childbirth.

**Figure 1:**
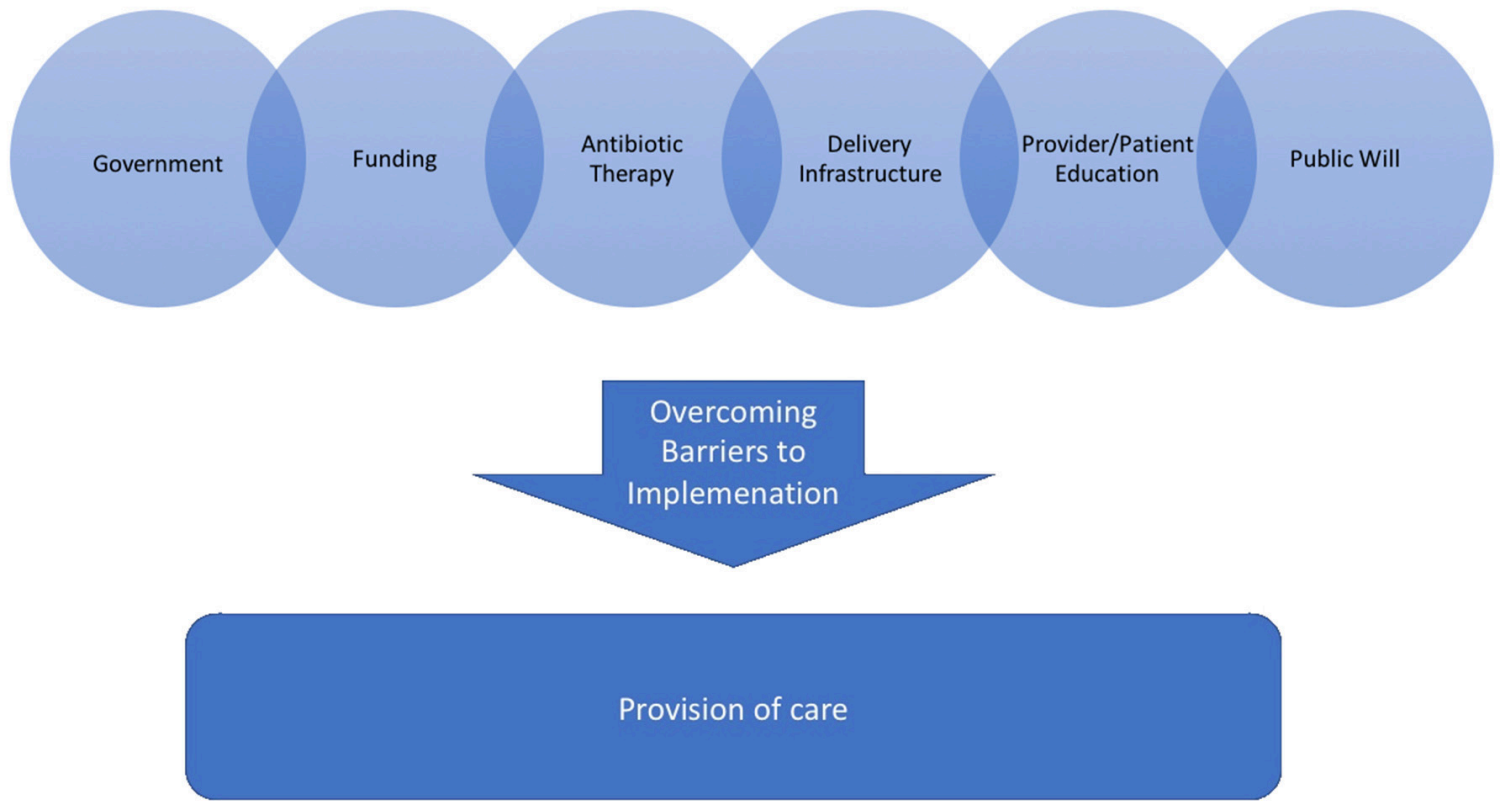
**A pictorial description of the factors that are needed for implementation of a policy for antibiotic therapy for chorioamnoninits into a society where it is otherwise not accessible**.

Attempts to create implementable regimens in various regions will rely on multiple factors for implementation (Figure 1). Evaluation of simplified regimens will be necessary to demonstrate effective policies but also to encourage widespread implementation of effective treatment modalities (Zaidi et al., 2013). Effective implementation of antibiotic therapy for chorioamnionitis will require continued success with Sustainable Development goals aiming to promote well-being for mothers and children. Public Health efforts to minimize the maternal and neonatal morbidity associated with chorioamnionitis will continue. As attention is directed toward areas where treatment is not currently available, those pregnancies in low resource settings will benefit. As much focus as will need to be put on birth attendance and appropriate delivery hygiene, provision of appropriate antibiotic therapy for chorioamnionitis will remain important.

## Conclusions

Various antibiotics and classes of antibiotics may be considered for effective treatment of chorioamnionitis. Ethical demonstration of benefit is problematic, given that a number of agents have long been considered effective therapy (Higgins et al., 2016). We outline a number of different antibiotic types that might be considered when developing treatment algorithms for chorioamnionitis, particular in LMIC were current treatment is far less than what a population might need to minimize maternal and neonatal complications. Effective treatment of chorioamnionitis will require more than just antibiotic therapy; it will require a birth companion competent to diagnose and treat the condition, an infrastructure to provide such antibiotics to the appropriate providers. As global efforts to increase the safety of birth advance, there is an urgent need for antibiotic therapy to appropriately manage chorioamnionitis in order to optimize maternal and neonatal outcomes.

## Author contributions

CJ, RA, and AF: Substantial contributions to the conception or design of the work; or the acquisition, analysis, or interpretation of data for the work; and drafting the work or revising it critically for important intellectual content; Final approval of the version to be published; Agreement to be accountable for all aspects of the work in ensuring that questions related to the accuracy or integrity of any part of the work are appropriately investigated and resolved.

### Conflict of interest statement

The authors declare that the research was conducted in the absence of any commercial or financial relationships that could be construed as a potential conflict of interest. The reviewer MLR and handling Editor declared their shared affiliation, and the handling Editor states that the process nevertheless met the standards of a fair and objective review.
